# Accuracy of Dynamic Navigation vs. Freehand Endodontic Access Cavity Preparation in 3-Dimensionally Printed Teeth with Severe Pulp Canal Calcification

**DOI:** 10.3390/jfb16100376

**Published:** 2025-10-09

**Authors:** Egle Marija Urbone, Paulius Tusas, Ieva Gendviliene, Vygandas Rutkunas, Saulius Drukteinis

**Affiliations:** Institute of Odontology, Faculty of Medicine, Vilnius University, Žalgirio Str. 115, LT-08217 Vilnius, Lithuania

**Keywords:** calcified canals, pulp canal calcification, dynamic navigation, guided endodontics, accuracy, cone-beam computed tomography, endodontic access cavity, dynamic computer-assisted navigation, real-time navigation, root canal treatment

## Abstract

Background: Pulp canal calcification (PCC) poses a challenge for endodontic treatment, as it obscures the canal and increases the risk of complications. This study aimed to evaluate the accuracy of endodontic access cavity preparation using dynamic navigation (DN) and to compare it with the freehand (FH) technique in teeth with severe PCC. Materials and Methods: Sixty 3D printed maxillary central incisors with simulated severe PCC were divided into two groups and accessed either with a DN system or by the conventional FH technique. Accuracy was evaluated by comparing planned and performed access cavity trajectories on preoperative and postoperative CBCT scans. Preparation time and procedural errors were recorded. Normality was assessed with the Shapiro–Wilk test. The Mann–Whitney U test was used to compare continuous variables. The significance level was set at 0.05. Results: The DN group showed significantly lower apical point 3D deviation (1.25 vs. 1.96 mm, *p* = 0.001), apical point depth deviation (0.43 vs. 0.88 mm, *p* < 0.001), and angular deflection (1.93 vs. 5.71 degrees, *p* < 0.001) than the FH group. The DN group had fewer procedural errors. The endodontic access entry point deviation was comparable between both techniques (*p* = 0.395). The preparation time was significantly higher in the DN group (204 vs. 108.5 s, *p* < 0.001). Conclusions: DN significantly improves the accuracy of access cavity preparation in calcified canals compared to the FH approach, reducing the risk of complications. Therefore, DN can be a valuable tool for managing challenging endodontic cases. As guided endodontic access preparation can be more time-consuming, extended treatment appointments may be required.

## 1. Introduction

Pulp canal calcification (PCC), also known as pulp canal obliteration or calcific metamorphosis, is a process of hard tissue formation and deposition within the pulp space, resulting in a reduced volume of the pulp cavity and root canals. PCC commonly occurs following dental trauma [1], deep caries or dental restorations [2], age-related dentine apposition, particularly in elderly patients [3]. It may also be associated with systemic illnesses [4], and long-term use of medications [5]. Recently, PCC has become increasingly recognized as a common complication following regenerative endodontic treatment and vital pulp therapy. The incidence of PCC has been reported to be 78% after regenerative endodontic procedures [6]. As vital pulp therapies gain popularity in clinical practice, the incidence of PCC is expected to rise among young patients.

Although PCC alone is generally not an indication for root canal treatment [7], up to 33% of calcified teeth may eventually present clinical and radiographic evidence of pulpal or periapical pathology [8]. The endodontic treatment of teeth with PCC is associated with an increased risk of complications, such as missed canals, canal transportation, crown or root perforations, and compromised tooth structure [9,10]. Additionally, PCC can significantly extend the time required for canal location, sometimes up to 60 min [3]. To overcome these challenges, a variety of treatment strategies for managing teeth with PCC have been proposed in the literature. These include the use of a dental operating microscope (DOM) [11], ultrasonic tips, performing the sodium hypochlorite “bubble test” [12], positioning the access cavity closer to the incisal edge in anterior teeth [1], and taking multiple-angle radiographs during endodontic access preparation [13]. To further improve the efficacy and accuracy of endodontic access preparation, the concept of guided endodontics has emerged [14]. Guided endodontics includes both static and dynamic navigation techniques, which may assist clinicians in accurately locating calcified root canals [15,16,17,18].

The workflow of static navigation is based on the principle of guided implantology and implies the use of 3D printed template with an incorporated sleeve that guides the bur during endodontic access cavity preparation. However, this approach has several limitations, including prolonged planning time, potential inaccuracies in preoperative CBCT or intraoral scanning, challenging application in premolar and molar regions due to limited vertical space, and the necessity for straight-line access to the root canal. Consequently, the use of static guides is largely confined to anterior teeth. Moreover, treatment typically cannot be completed in a single visit due to the need for 3D printing of the template, and the costs for the patient are also increased [15].

Dynamic navigation (DN) systems utilize specialized software for preoperative planning of endodontic access cavities. A motion-tracking camera detects markers attached to both the patient and the dental handpiece, enabling real-time visualization of the drill’s position and trajectory across multiple sectional planes within a three-dimensional imaging dataset. This ensures adherence to the preoperative plan. DN offers several advantages over static guides: it allows adjustments to the drilling path through real-time tracking, it can be used in posterior teeth, and enables treatment to be completed within a single appointment [15]. Early experimental studies have demonstrated the promising accuracy of DN in endodontic access cavity preparation [19]. However, recent clinical reports have provided conflicting findings [20,21,22,23,24]. In addition, previous in vitro studies have often lacked standardized outcome measures and adequate sample sizes [19]. Consequently, further in vitro evidence is necessary to thoroughly validate the accuracy and reliability of DN for endodontic access preparation.

This study aimed to evaluate the accuracy, procedural time, and incidence of procedural mishaps during endodontic access cavity preparation in severely calcified root canals using dynamic navigation (DN), and to compare these outcomes with the conventional freehand (FH) technique. The null hypothesis was that there is no significant difference in accuracy between the DN and FN techniques.

## 2. Materials and Methods

### 2.1. Sample Size Calculation

A priori G*power Software (version 3.1, Germany) analysis for difference between two independent means was performed to determine the required sample size. Based on previously reported effect sizes in similar studies [16], the minimum sample size needed to achieve a statistical power of at least 0.8 with an alpha of 0.05 and an effect size (d = 0.71) was determined to be 26 teeth per group. To ensure reliability and account for possible procedural errors, a sample size of 30 teeth per group was selected.

### 2.2. Teeth Selection

**Figure 1:**
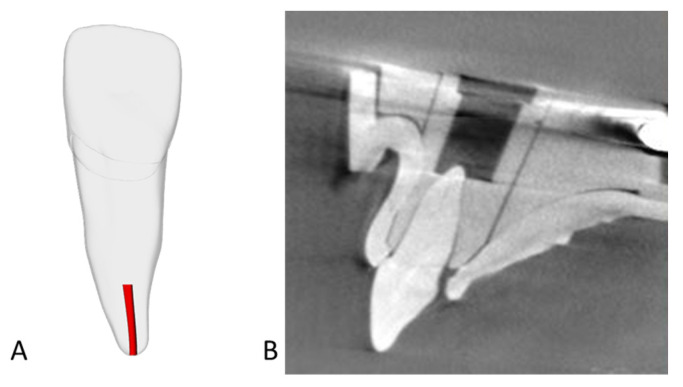
Three-dimensional printed single-rooted maxillary central incisors (DRSK Group AB, Hässleholm, Sweden). (**A**)—Schematic representation of the 3D-printed teeth with simulated severe PCC (canal marked in red) [25]; (**B**)—CBCT image of the 3D-printed teeth exhibiting simulated severe PCC.

Sixty 3D printed single-rooted maxillary central incisors (DRSK Group AB, Hässleholm, Sweden) were selected. These teeth had simulated severe PCC, with root canals present only in the apical portion of the root (5 mm from the root apex), and similar radiopacity to human teeth (Figure 1). The teeth were randomly allocated into two groups: the dynamic navigation (DN) group (*n* = 30), and the freehand (FH) group (*n* = 30).

Teeth were mounted onto a modular jaw (DRSK Group AB, Hässleholm, Sweden) using a flowable gingival barrier (BEYOND BlueSeal Gingival Protection, Sugar Land, TX, USA) to ensure tooth stability during the experimental procedures.

### 2.3. Planning

A preoperative single-arch high-resolution CBCT scan was taken using a KaVo OP 3D scanner (KaVo Dental GmbH, Biberach an der Riß, Germany) at a voxel size of 0.200 mm. The acquired DICOM dataset was imported into the Navident (v4.1.0, ClaroNav, Toronto, ON, Canada) planning software. The calcified canal was identified, and a straight endodontic access cavity of 0.8 mm diameter and 14.5 mm depth from the starting point on the palatal tooth surface (19 mm from the incisor edge) was planned.

The modular upper jaw was mounted into a dental manikin using PreVISION Temp material (Kulzer GmbH, Hanau, Germany). All procedures were conducted by a single operator, a board-certified endodontist with 5 years of clinical experience, who received specialized DN training from the manufacturer, and had completed 20 procedures before the study.

Access cavities were prepared using an Edenta Endo bur (Edenta AG, Au, Switzerland) measuring 0.8 mm in diameter and 34 mm in length, mounted on a slow-speed handpiece set to a maximum of 5000 RPM. A new bur was used for each tooth.

### 2.4. DN Access Cavity Preparation

**Figure 2:**
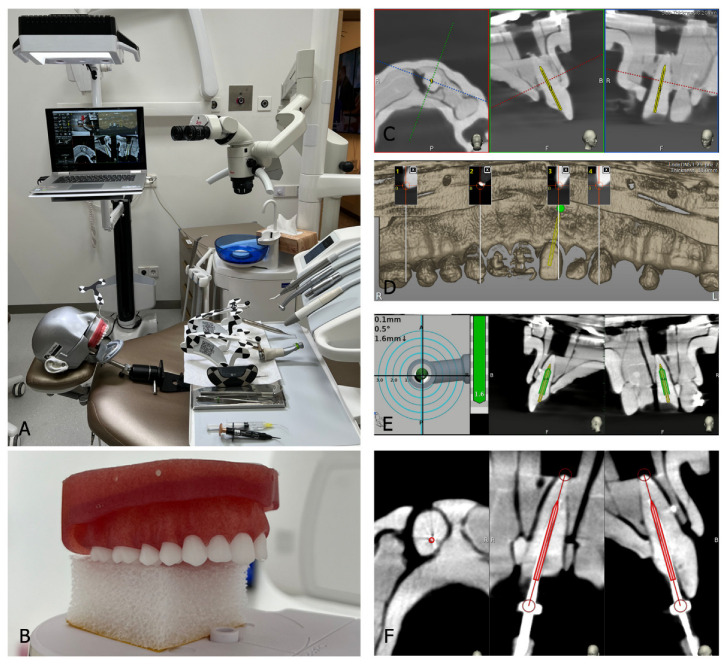
The Navident workflow. (**A**)—a setup of the DN system; (**B**)—a pre-operative CBCT scan of the model is acquired; (**C**)—the CBCT scan is imported in Navident and the virtual endodontic access cavity is planned (marked in yellow); (**D**)—the tracing and the accuracy check is performed; (**E**)—the endodontic access cavity is being drilled following the “target” views on the computer screen (actual drilling path marked in green); (**F**)—a post-operative CBCT scan of the model is acquired and the prepared endodontic access cavity is evaluated (prepared cavity marked in red).

Access cavities were prepared using dynamic navigation with the Navident II/UNO system (ClaroNav, Toronto, ON, Canada), along with its JawTracker and DrillTag components (Figure 2). The JawTracker was bonded to the maxillary teeth with flowable composite resin, and the DrillTag was secured to a slow-speed handpiece. Tracer tool calibration and trace registration were performed at four anatomically distinct non-collinear points, followed by drill axis and drill tip calibration. Accuracy was verified on the buccal, incisal, and palatal surfaces, with all steps repeated if deviation exceeded 0.5 mm. During access preparation, the drill tip was aligned and its trajectory continuously monitored in real time using the preoperative plan displayed in the target view of the Navident software. Drilling was terminated upon reaching the planned endpoint. The total procedure time, including planning, tracing, calibration, and drilling, was recorded. Actual drilling time was measured separately using a stopwatch.

### 2.5. FH Access Cavity Preparation

Freehand access cavities were prepared using a DOM (Leica M320, Leica Microsystems GmbH, Wetzlar, Germany). Preoperative CBCT scans were available to aid in the creation of access cavities. Drilling was performed to a depth of 19 mm, measured from the incisal edge. Total procedure time was recorded using a stopwatch. To prevent the operator from memorizing tooth anatomy, treatment sessions were scheduled at intervals of at least three days, and each tooth was mounted in the modular jaw with a varied angulation.

### 2.6. Accuracy Determination

**Figure 3:**
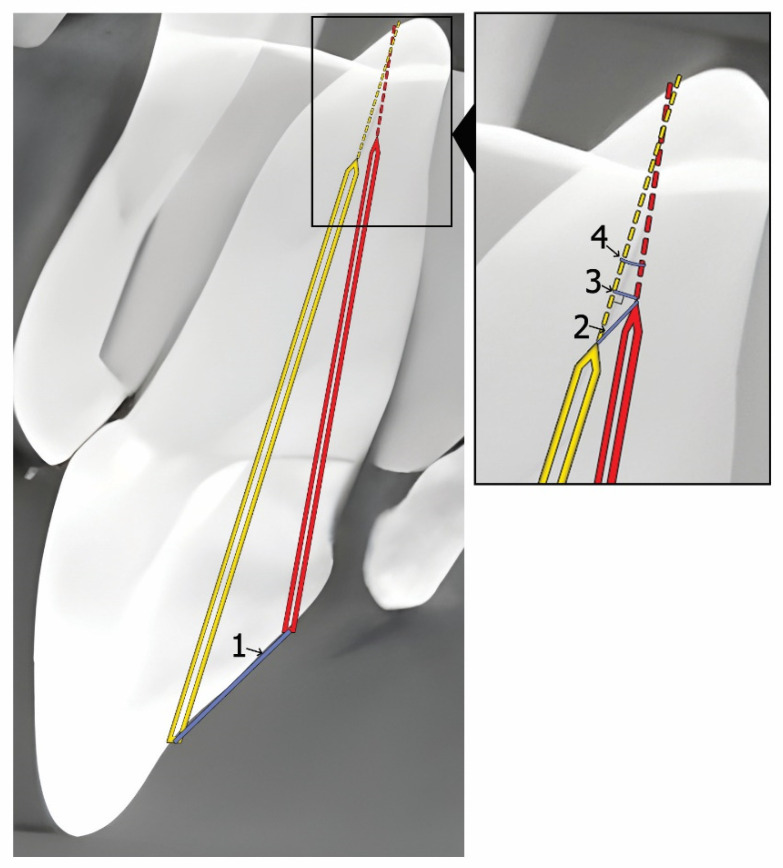
A schematic view of accuracy analysis. 1—entry point deviation (mm); 2—apical point 3D deviation (mm); 3—apical point depth deviation (mm); 4—angular deflection (degrees).

Accuracy evaluation was conducted by two blinded, independent researchers, both of whom are board-certified endodontists with five and seven years of experience. Following preparation, a #10 K-file was inserted into each access cavity, and a postoperative CBCT scan was taken using identical exposure parameters. Preoperative and postoperative CBCT scans were superimposed using the EvaluNav software (v1.4.0) application (included in Navident). The following parameters were assessed for accuracy (Figure 3):

- Endodontic access entry point deviation (mm): the linear deviation at the coronal entry point.
- Apical point 3D deviation (mm): the total linear deviation at the apical endpoint.
- Apical point depth deviation (mm): the vertical deviation measured at the apical endpoint.
- Angular deflection (degrees): the maximal angular difference between the planned and actual cavity trajectory.

Additionally, operation times were compared between groups, and root perforations were assessed through direct visual inspection.

### 2.7. Statistical Analysis

Statistical analysis was performed using IBM SPSS Statistics version 30.0. Two observers obtained measurements, and the average value was used for analysis. Agreement between the two observers was assessed using the intraclass correlation coefficient (ICC). ICC estimates and their 95% confidence intervals were calculated based on a mean-rating (k = 2), absolute-agreement, 2-way mixed-effects model. The Shapiro–Wilk test was applied to assess the normality of variable distributions. Given the non-normal distribution, continuous variables were presented as medians with interquartile range (IQR). Continuous variables were compared using the Mann–Whitney U test for two groups and the Kruskal–Wallis test for three or more groups. When the Kruskal–Wallis test was significant, pairwise comparisons were conducted using Dunn’s test with Bonferroni adjustment. The Chi-square test was used to assess the difference in perforation frequency between the DN and FH groups. To evaluate the learning curve, a cumulative sum (CUSUM) analysis was performed. Cases were ordered chronologically from first to last, and CUSUM values were computed as follows:

$$Si=\;\sum_{\left\{j=1\right\}}^{i}(x_{j}-\;\mu )$$

where *x_j_* is the endodontic access preparation time for case *j* and *μ* is the mean preparation time in the DN group. A two-sided *p*-value < 0.05 was considered statistically significant. Figures were created using GraphPad Prism (version 10.6).

## 3. Results

### 3.1. Agreement Between Measurements

The inter-rater reliability was excellent (ICC = 0.98; 95% CI: 0.96–0.99).

### 3.2. Accuracy

Median values of endodontic access entry point deviation, apical point 3D deviation, apical point depth deviation, and angular deflection are summarized in Table 1. The entry point deviations were similar in both groups (*p* = 0.395). However, significantly lower deviations were observed in the DN group for apical 3D deviation (*p* = 0.001), apical depth deviation (*p* < 0.001), and angular deflection (*p* < 0.001) compared to the FH group (Table 1).

### 3.3. Procedural Time and Mishaps

**Figure 4:**
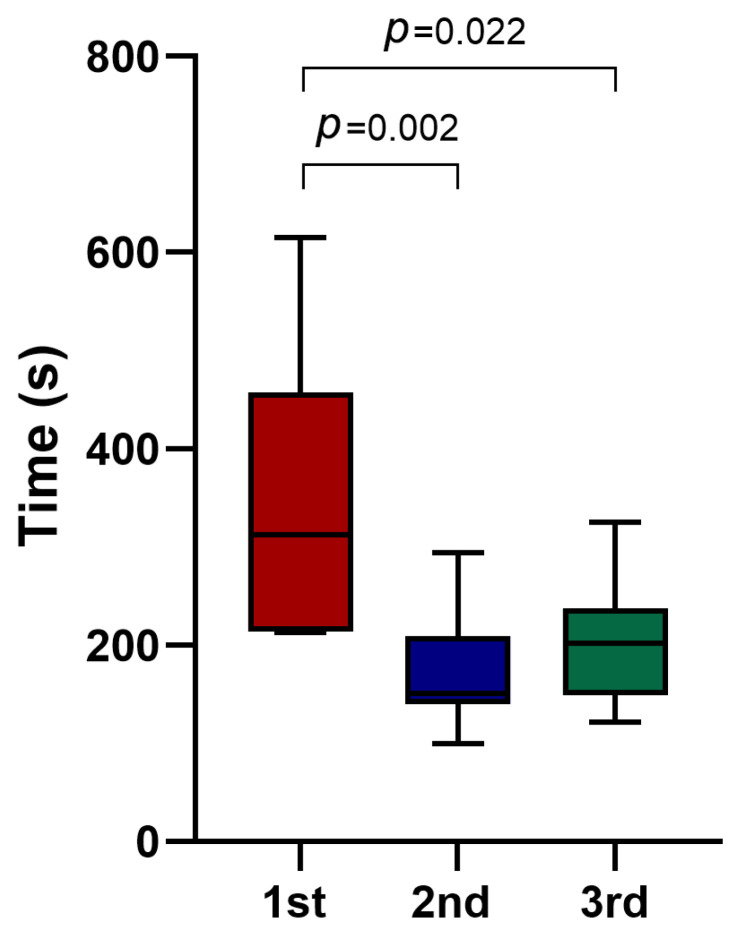
Endodontic access cavity preparation time across the thirds in the DN group.

The median time of the endodontic access preparation was significantly lower in the FH group (*p* < 0.001) (Table 2). Within the DN group the first 10 cases required longer endodontic access preparation time compared to time required for the second 10 cases (312 s [IQR 214–457] vs. 151 s [IQR 140–209.25], *p* = 0.002) and compared to time required for final 10-case blocks preparation (312 s [IQR 214–457] vs. 202 s [IQR 149.25–237], *p* = 0.022) (Figure 4). By contrast, in the FH group median times did not differ significantly across the three 10-case blocks—114 s (IQR 98.75–148.5), 113.5 s (IQR 91.25–127), and 100 s (IQR 93.5–111.25), respectively (first vs. second: *p* = 0.533; first vs. final: *p* = 0.121; second vs. final: *p* = 0.354). The complete DN procedure (including planning, tracing, calibration, and drilling) required a median of 12 min. There were fewer root perforations in the DN group (*n* = 3) compared to the FH groups (*n* = 9), though this difference did not reach statistical significance (*p* = 0.053) (Table 2).

### 3.4. CUSUM Analysis

The learning curve was evaluated by plotting the outcomes in the CUSUM curve. The initial segment of the curve (Phase I), characterized by a progressive upward slope, reflects the operator’s learning phase, during which technical skills are being acquired and gradually refined. The subsequent plateau observed in the curve (Phase II) indicates the acquisition of a skill level sufficient for the consistent and reliable execution of the DNS procedure. A subsequent change in slope beyond this plateau (Phase III) indicates the point at which the operator has effectively surpassed the learning process, marking the consolidation of expertise.

**Figure 5:**
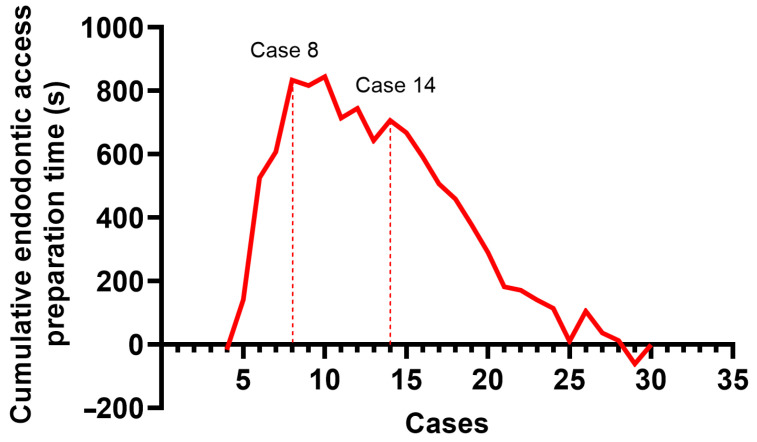
CUSUM analysis for endodontic access preparation time in the DN group.

For endodontic access cavity preparation time in the DN group two distinct peaks were identified at the 8th and 14th cases, delineating three phases: Phase I (case 1–8), Phase II (case 8–14), and Phase III (case 14–30) (Figure 5). CUSUM analysis for accuracy parameters in the DN group did not show distinct peaks.

## 4. Discussion

This study compared the accuracy, procedural time, and mishaps of DN with the FH technique for endodontic access preparation in teeth with severely calcified root canals. All canals were standardized and located at a depth of 19 mm from the incisal edge. DN resulted in increased accuracy and a lower number of root perforations, though the procedure required more time compared to FH.

To determine the accuracy of the DN and FH techniques, we compared the planned and obtained endodontic access cavities by superimposing the pre- and post-operative CBCT scans. The endodontic access entry point deviation, the 3D deviation of the apical point, the depth deviation of the apical point, and the angular deflection were calculated. The most critical parameters are the 3D deviation of the apical point and the angular deflection. In severe PCC cases, the angular deflection is closely linked to the apical point deviation, with greater angular deflection leading to the risk of root perforation and missed canals. In this study, the DN group showed superiority over the FH technique in both the apical point 3D deviation (*p* = 0.001) and the angular deflection (*p* < 0.001). This subsequently resulted in a lower number of root perforations in the DN group. However, using DN still led to three perforations. These errors can be attributed to the cumulative effects of inaccuracies in planning, calibration, and tracing, as well as JawTag movement, hand tremors, operator fatigue, and difficulty maintaining the drill tip at the center of the ‘target’ due to the considerable depth of the access cavity. In our study, a 3.73-degree angular deflection followed by a 2 mm apical point 3D deviation led to a perforation. To minimize the risk of root perforation, we recommend using a DOM and intraoperative radiographs as supplementary guidance during the use of DN.

Previous in vitro studies investigating the accuracy of DN for endodontic access cavity preparation in teeth with PCC have reported variable outcomes. Torres et al. [26] used anteriors, premolars, and molars to determine the outcome of using DN. The mean length of access cavities in anterior teeth was 13.04 mm (9.59–17.12 mm). The median of the endodontic access entry point deviation was 0.6 mm, the apical point deviation was 0.58 mm, the apical point depth deviation was 1.08 mm, and the angular deflection was 2.6 degrees. As the angular deflection was comparable with the one obtained in our study, the smaller apical deviation could be attributed to the shorter drilling path. Conversely, Huth et al. [27] reported higher deviations compared to our results, although the angular deviations remained comparable. The study compared DN with FH in four different anatomically challenging 3D printed replica teeth, including upper right canine with one root canal obliterated till the middle apical third. Their results revealed the median of the entry point deviation was 1.7 mm, the apical point deviation was 1.59 mm, and the angular deflection was 2.65 degrees. In another study, Jain et al. [15] used 3D-printed three identical sets of maxillary and mandibular jaw models, composed of 84 teeth, of which 36 were anterior teeth, to evaluate DN accuracy in locating simulated calcified canals. The average canal orifice depth was 12.4 mm. This study presented mean deviations without medians, complicating direct comparisons. Yet, their angular deviations were slightly lower: the mean endodontic access entry point deviation was 1.1 ± 0.8 mm, the mean apical point deviation was 1.3 ± 0.65 mm, the mean apical point depth deviation was 1 ± 0.65 mm, and the mean angular deflection was 1.7 ± 0.98 degrees. These values possibly reflect methodological differences, such as shorter drilling depths.

In a clinical setting, Sharma et al. [28] performed endodontic access cavity preparations on 45 calcified maxillary anterior teeth, reporting a mean angular deflection of 3.21 ± 2.23 (median 1.95) degrees and apical deviation of 0.52 ± 0.33 mm (median 0.45 mm). However, when analyzing teeth with PCC extending to the apical third of the root, the mean angular deflection increased to 4.3 ± 3.39 degrees, and the apical deviation to 0.85 ± 0.45 mm. These findings are in agreement with our results, suggesting that increased case complexity reduces accuracy, even when DN is applied.

In recent years, several case reports have described the successful localization of canals in teeth with PCC using DN [20,21,22,23,24,29]. Most of these cases involve maxillary and mandibular incisors; however, Dianat et al. reported successful localization of the distobuccal canal in a maxillary first molar [29]. Only one case report assessed the accuracy of DN, reporting the angular deflection of 4 degrees [20]. Conversely, Yang et al. reported two instances of failure to locate canals using DN, both involving deviated drill paths from the long axis of the tooth [22]. While multiple successes have been documented, some failures emphasize the critical importance of precise preoperative planning and careful execution.

DN was initially introduced in dentistry for dental implant placement, prompting numerous studies to evaluate its accuracy in dental implantology both in vitro and in clinical settings. A systematic review and meta-analysis, which included twenty-four in vitro and clinical studies, revealed mean overall angular deflections were 2.01 degrees (95% CI: 1.95 to 2.07) in in vitro studies and 3.68 degrees (95% CI: 3.61 to 3.74) in clinical studies [30]. These results are similar to those previously described in endodontology studies, indicating higher DN inaccuracies in clinical settings. Therefore, the need for cautious clinical implementation is emphasized.

The other Guided Endodontics method used for locating calcified canals is static navigation (SN). This technique uses 3D printed templates, which are planned using preoperative CBCT and intraoral scanning data [14]. Several studies compared DN with SN for endodontic access cavity preparation, reporting conflicting outcomes compared to DN [27,31,32]. Huth et al. found SN to be statistically significantly more accurate than DN [27]. On the contrary, Franco et al. reported SN to be less accurate than DN with an angular deflection of up to 29 degrees [31]. These differences can be attributed to many planning and production stages or ill-fitting SN guides. The accuracy measures of the DN group were comparable in both studies. Recently, Torres et al. compared SN with the FH technique in a clinical study [18]. Their findings demonstrated that the SN group achieved statistically significantly better outcomes, with fewer technical complications such as missed canals or perforations. Despite these advantages, the authors emphasized that SN remains a technically demanding procedure that should be performed by experienced endodontists with the aid of DOM. Variations in results may be attributable to multiple procedural factors and inconsistencies in guide fitting accuracy, highlighting the continued importance of operator expertise.

The duration of endodontic treatment is an important consideration for both patients and clinicians. In our study the complete DN procedure required a median of 12 min, which is comparable to the findings of Hildebrand et al., who reported a mean DN procedure time of 13.7 min [33]. The median preparation time for drilling alone was significantly longer in the DN group compared with the FH technique. This finding is consistent with Huth et al. [27], although other studies have reported contrasting results [16,17]. Differences in reported procedure times across studies may be attributed to variations in measurement start and endpoints, simulated clinical conditions, and methodological differences. Moreover, although the operator had prior experience with the DN before the experiments, the first third still required significantly more time compared with the second and third thirds. This finding suggests that each new clinical situation demands additional time. Given that guided endodontic access preparation can be more time-consuming, longer treatment appointments may be necessary. An additional consideration regarding the use of DN is the increased treatment cost, as clinics must invest in specialized hardware, software, and operator training. These costs may, in turn, increase the overall treatment price for patients, potentially limiting the widespread adoption of DN technology in routine endodontic practice.

As the primary aim of our study was to evaluate the accuracy parameters of DN, achieving a high degree of standardization was essential. Therefore, we selected 3D-printed teeth, which allowed us to replicate a particularly challenging clinical scenario while ensuring a high level of standardization, comparability, and an adequate sample size per group. Previous research on DN in endodontics has typically focused on partially calcified canals, shallower depths, or variable anatomy, limiting their relevance to the most demanding cases [15,26,27]. 3D-printed teeth, however, lack the natural variations in dentin color and consistency that may assist clinicians in locating canals using magnification. However, in cases where canals exist only in the apical third of the root, the use of a DOM becomes less effective due to the considerable depth of the endodontic access. As a result, the FH technique relies primarily on selecting the correct angulation to align the bur with the long axis of the tooth. To minimize operator bias and avoid memorization of the tooth anatomy, experimental sessions were scheduled at least three days apart. In addition, the angulation of each tooth was systematically altered when mounting it in the modular jaw, thereby preventing reliance on previously learned bur angulations during drilling.

Studies show that working with the DN system can be challenging for novice operators [26,27]. The practitioner should be comfortable with drilling while looking at the computer screen for visual guidance. To minimize the impact of the learning curve and operator-related errors, the operator received training from Navident on the use of the DN system and completed 20 endodontic access preparations on teeth with varying anatomies prior to the experiment. Furthermore, a CUSUM analysis was performed to assess a potential learning curve during the experiment. The analysis showed no distinct peaks in the accuracy parameter curves, indicating stable performance. These findings are consistent with previous studies, which recommend completing 20 cases prior to experimentation to overcome the initial learning phase [26,28]. In contrast, the curve of endodontic access cavity preparation time in the DN group displayed three distinct phases, suggesting that each new clinical scenario may require additional time to master.

Beyond its clinical applications, DN may offer substantial educational value. Studies investigating the use of DN to train students in dental implant placement reported high levels of student satisfaction and demonstrated that DN assisted training significantly improved placement accuracy [34,35]. The real-time feedback provided by DN on drill angulation, depth, and trajectory has the potential to accelerate the development of three-dimensional spatial reasoning and endodontic access skills—particularly in complex cases such as teeth with PCC. When integrated with a DOM, DN enables students to correlate the operative field with sectional imaging in real time, thereby enhancing their understanding of root canal anatomy and improving hand–eye coordination during access preparation. Future studies should examine the integration of DN into endodontic education and its impact on learning outcomes.

This study has several limitations that should be acknowledged. First, the single-operator study design may have introduced bias, potentially overestimating the performance and accuracy of the technique. As the operator was experienced and specifically trained for the procedure, the results may not be fully generalizable to clinicians with different levels of experience or training. Secondly, while the use of standardized in vitro 3D-printed models allowed for controlled anatomical conditions and ensured adequate statistical power, they do not fully replicate the optical and tactile properties of natural dentin. Moreover, the model lacked critical clinical variables such as patient movement, restricted intraoral access, and interference from soft tissues. As a result, the experimental environment may not accurately reflect the complexities encountered in real clinical scenarios. A further limitation of this study is the exclusive use of standardized maxillary central incisors with straight, single canals and a direct access trajectory to the canal orifice. While this approach facilitated consistency and control across samples, it does not reflect the anatomical complexity encountered in clinical practice, particularly in teeth with curved canals, multiple roots, or more challenging posterior locations. In such cases, the accuracy of the technique may differ.

This study demonstrates that DN significantly enhances accuracy and reduces the risk of perforation compared to the FH technique. Nevertheless, we advise caution when using DN in the management of complex PCC cases. The use of a DOM and intraoperative radiographs, combined with DN, could further increase accuracy in clinical settings. Further in vitro studies could investigate the combined use of DN and DOM to enhance precision during endodontic access cavity preparation. Given that DN demonstrated reliable and predictable outcomes in highly standardized 3D-printed models, subsequent studies should also focus on natural teeth with variable anatomy to better reflect clinical realities. Moreover, including multiple operators with varying levels of experience would allow for a more comprehensive evaluation of the learning curve and help determine the reproducibility of the technique across different clinical settings. Ultimately, in vivo studies will be essential to validate the performance and clinical applicability of DN under real-world conditions.

## 5. Conclusions

In this in vitro study, dynamic navigation provided significantly greater accuracy than the freehand technique in endodontic access preparations of severely calcified canals. Although dynamic navigation required longer preparation times, it resulted in fewer root perforations. Dynamic navigation can be a valuable tool for managing challenging endodontic cases. Since guided endodontic access preparation may require more time, longer treatment appointments might be necessary.

## Figures and Tables

**Table 1:** Accuracy measurements.

Accuracy Measurements	DN Group	FH Group	*p* Value
Entry point deviation, mm	0.60 (0.38–1.06)	0.55 (0.36–0.79)	0.395
Apical point 3D deviation, mm	1.25 (0.71–1.49)	1.96 (1.43–2.21)	0.001
Apical point depth deviation, mm	0.43 (0.22–0.62)	0.88 (0.54–1.46)	<0.001
Angular deflection, degrees	1.93 (1.49–3.54)	5.71 (3.67–8.16)	<0.001

Data is provided in medians (IQR). DN—dynamic navigation, FH—freehanded.

**Table 2:** Procedural time and root perforations.

Efficiency Measurements	DN Group	FH Group	*p* Value
Median time, s	204 (148–291)	108.5 (96.25–127)	<0.001
Mean time, s	230.3 ± 114.92	110 ± 23.48	<0.001
Minimum time, s	100	63	N/A
Maximum time, s	615	160	N/A
Perforation, *n* (%)	3 (10.0)	9 (30.0)	0.053

DN—dynamic navigation, FH—freehanded.

## Data Availability

The original contributions presented in the study are included in the article, further inquiries can be directed to the corresponding authors.

## Abbreviations

The following abbreviations are used in this manuscript:

CBCT	Cone Beam Computed Tomography
DN	Dynamic Navigation
FH	Freehanded
DOM	Dental Operating Microscope
ICC	Intraclass Correlation Coefficient
3D	Three-dimensionally
PCC	Pulp Canal Calcification
SN	Static Navigation
